# Social-Cyber Maneuvers During the COVID-19 Vaccine Initial Rollout: Content Analysis of Tweets

**DOI:** 10.2196/34040

**Published:** 2022-03-07

**Authors:** Janice T Blane, Daniele Bellutta, Kathleen M Carley

**Affiliations:** 1 School of Computer Science Carnegie Mellon University Pittsburgh, PA United States

**Keywords:** social cybersecurity, social-cyber maneuvers, social network analysis, disinformation, BEND maneuvers, COVID-19, coronavirus, social media, vaccine, anti-vaccine, pro-vaccine, ORA-PRO, cybersecurity, security, Twitter, community, communication, health information, manipulation, belief

## Abstract

**Background:**

During the time surrounding the approval and initial distribution of Pfizer-BioNTech’s COVID-19 vaccine, large numbers of social media users took to using their platforms to voice opinions on the vaccine. They formed pro- and anti-vaccination groups toward the purpose of influencing behaviors to vaccinate or not to vaccinate. The methods of persuasion and manipulation for convincing audiences online can be characterized under a framework for social-cyber maneuvers known as the BEND maneuvers. Previous studies have been conducted on the spread of COVID-19 vaccine disinformation. However, these previous studies lacked comparative analyses over time on both community stances and the competing techniques of manipulating both the narrative and network structure to persuade target audiences.

**Objective:**

This study aimed to understand community response to vaccination by dividing Twitter data from the initial Pfizer-BioNTech COVID-19 vaccine rollout into pro-vaccine and anti-vaccine stances, identifying key actors and groups, and evaluating how the different communities use social-cyber maneuvers, or BEND maneuvers, to influence their target audiences and the network as a whole.

**Methods:**

COVID-19 Twitter vaccine data were collected using the Twitter application programming interface (API) for 1-week periods before, during, and 6 weeks after the initial Pfizer-BioNTech rollout (December 2020 to January 2021). Bot identifications and linguistic cues were derived for users and tweets, respectively, to use as metrics for evaluating social-cyber maneuvers. Organization Risk Analyzer (ORA)-PRO software was then used to separate the vaccine data into pro-vaccine and anti-vaccine communities and to facilitate identification of key actors, groups, and BEND maneuvers for a comparative analysis between each community and the entire network.

**Results:**

Both the pro-vaccine and anti-vaccine communities used combinations of the 16 BEND maneuvers to persuade their target audiences of their particular stances. Our analysis showed how each side attempted to build its own community while simultaneously narrowing and neglecting the opposing community. Pro-vaccine users primarily used positive maneuvers such as excite and explain messages to encourage vaccination and backed leaders within their group. In contrast, anti-vaccine users relied on negative maneuvers to dismay and distort messages with narratives on side effects and death and attempted to neutralize the effectiveness of the leaders within the pro-vaccine community. Furthermore, nuking through platform policies showed to be effective in reducing the size of the anti-vaccine online community and the quantity of anti-vaccine messages.

**Conclusions:**

Social media continues to be a domain for manipulating beliefs and ideas. These conversations can ultimately lead to real-world actions such as to vaccinate or not to vaccinate against COVID-19. Moreover, social media policies should be further explored as an effective means for curbing disinformation and misinformation online.

## Introduction

### Background & Motivation

COVID-19 claimed the lives of 2.6 million people in the first year of its discovery [1]. In a concerted effort to reduce the cases and deaths resulting from the COVID-19 pandemic, governments and major health organizations pushed for the development and rapid distribution of COVID-19 vaccines. This process, however, has been met with online expressions of resistance vaccination [2]. In view of this concerning spread of anti-vaccine sentiment online, our work has focused on identifying the specific tactics used by both the pro- and anti-vaccine communities to spread their messages over Twitter.

Though vaccinating everyone against COVID-19 may seem to be an obvious way to prevent deaths, many people and groups oppose vaccination for several different reasons. The first compulsory vaccination was established in England by the Vaccination Act of 1853. The act faced opposition to the idea that the government should impose health legislation [3]. In current times, communities speak out against the government and assert that they have the right to decide what goes inside their bodies. Some anti-vaccine proponents fear the side effects of vaccines and refuse entirely to vaccinate themselves or their children because of rumors of autism or other medical disorders [4,5].

The Pfizer-BioNTech vaccine was the first vaccine for preventing COVID-19 to be authorized in the United States by the Federal Drug Administration (FDA). Both the FDA and European Medicines Agency (EMA) authorized the vaccine for emergency use. In 2020, the first Pfizer vaccine doses were distributed in the United Kingdom on December 8 and in the United States on December 14. Because of the rush to create the vaccine, many feel the vaccines were inadequately tested and refuse the vaccine without seeing the results of long-term studies. Additionally, some accept conspiracy theories or rumors on the vaccine. For example, one such conspiracy theory is that Bill Gates and the government created the vaccines to microchip the population for some malicious intent [6]. These are among the reasons for “vaccine hesitancy” across the world [7].

Social media has become a medium for COVID-19 vaccine discussion. Twitter is a popular platform on which government leaders, public health officials, and news organizations spread pertinent information. However, many users spread misinformation or act maliciously by conducting influence campaigns to manipulate peoples’ beliefs and ideas. Bonnevie et al [2] found that vaccine opposition on Twitter increased by 80% after COVID-19 began spreading in the United States. Misinformation is not limited to anti-vaccine users as some pro-vaccine users also share unreliable information [8]. To counter the spread of misinformation on its platform during the initial administration of the vaccine, Twitter expanded its policy by removing false and misleading tweets about COVID-19 vaccines, adding labels to potentially misleading COVID-19 vaccine information, and creating a “five-strike system” for suspending misleading accounts [9,10].

These malicious actions online are a major aspect within the field of social cybersecurity. Social cybersecurity lies at the intersection between cyberspace and human interaction. It studies how humans can be influenced by tactful messaging and connecting the right people to the right content. Key players in an online social network can conduct influence maneuvers to change users’ beliefs and affect their behavior [11]. In our study, we sought to identify the important actors in pro- and anti-vaccine Twitter communities as well as the social-cyber maneuvers they used to influence their audiences’ stances regarding the COVID-19 vaccine.

This work focused on the time period around the approval and initial administration of the Pfizer vaccine. Our objective was to determine whether there are differences between the types of social-cyber maneuvers pro-vaccine and anti-vaccine communities use toward their target audiences. We describe a methodology for determining pro-vaccine or anti-vaccine stances within tweets and identifying key players within the social network. We further used bot detection and linguistic cues to analyze the content and significance of tweets, and we evaluated how the 2 opposing vaccine communities applied social-cyber maneuvers to persuade their target audiences. Our results show how pro-vaccine messaging focused on exciting readers and explaining the vaccine issue. In contrast, anti-vaccine groups preferred to make dismaying statements and used messaging that distorted vaccine information. We also found that Twitter’s tightening of its policies on vaccine misinformation had a remarkable effect on decreasing the size of anti-vaccine communities and the prevalence of their messaging.

### Related Work

#### Vaccine Stance Detection

The problem of identifying pro- and anti-vaccine communities has garnered the attention of several researchers who have sought to apply stance detection techniques from computer science to this task. Supervised machine learning methods developed for this problem have ranged from the use of transformer neural networks based on Google’s Bidirectional Encoder Representations from Transformers (BERT) model [12] to the use of convolutional neural networks trained on n-grams and topics detected via Latent Dirichlet Allocation [13]. More traditional community detection algorithms have also been used to find groups with overt stances on vaccines [14]. The semisupervised stance propagation technique used for our work, which has the advantage of not requiring extensive manual labelling of pro- and anti-vaccine messages, was also used to identify linguistic differences between pro- and anti-vaccine groups [15].

#### Pro-Vaccine and Anti-Vaccine Communities

Various studies of anti-vaccine and pro-vaccine communities have sought to identify the methods used for spreading vaccine-related messages. Different communities can have contrasting messaging characteristics depending on the nature of and support for their stances on vaccines.

In 2019, a study examining influential themes and actors within the anti-vaccine community concluded that top tweeters relied on highly networked communities led by accounts that select messages expected to have high receptivity within those communities [16]. This was different from standard messages from public officials, which tended to repeat the same information to the same communities, limiting the extent of a message’s reach. In an analysis of Facebook vaccine group clusters, Johnson et al [17] observed that anti-vaccination clusters entangled more often with undecided clusters, while pro-vaccination clusters tended to be more peripheral. Furthermore, Schmidt et al [14] examined how echo chambers reinforce the opinions of groups and how involvement within these groups could be an effective way of countering anti-vaccine beliefs.

Past research has therefore found that pro-vaccine messages tend to be supported by public health officials and governments seeking to reduce the spread of infectious diseases, whereas anti-vaccine communities are more niche and maintain a smaller following. However, although pro-vaccine messages tend to stay within pro-vaccine communities, anti-vaccine messages permeate beyond the boundaries of anti-vaccine communities.

Although these past works have analyzed the themes and targeting of vaccine messaging, they have not considered the specific types of strategies carried out in vaccine-related information operations. Thelwall et al [18] tracked some of the anti-vaccine narratives spreading on Twitter, and Boucher et al [19] identified the key themes in Twitter conversations about vaccine hesitancy. However, previous research has not examined the intentions behind specific choices on the language, content, and targeting of pro- and anti-vaccine messaging. Our work breaks down the tactical value of specific types of vaccine messages and analyzes how those tactics have changed over time.

#### Social Cybersecurity: Influence Campaigns and Bots

A key development in the fight against online influence campaigns has been the growth of the field of social cybersecurity, a computational social science that aims to protect the security of democratic societies by studying the ways in which actors exercise manipulation on social media platforms [11]. Recognized by the National Academies as a new science [20], its key areas of research have been the study of information maneuvers, motive identification, and information diffusion, as well as the evaluation of the effectiveness of information campaigns and mitigation strategies. Though the field has most extensively focused on the spread of political disinformation, it has more recently expanded to tackle the problem of medical misinformation [11].

Of particular concern in social cybersecurity is the existence of automated accounts on social media platforms since they are used to spread online disinformation and influence elections [21]. They have also manipulated public health discourse by propagating misinformation on topics such as e-cigarettes, diets, and medications [22]. Because of the influence of these bots on public opinion, several studies have been conducted on the use of bots for spreading vaccine information [23,24]. Before the COVID-19 pandemic, Broniatowski et al [25] examined the extent to which bots spread anti-vaccine messages, showing the high rates of vaccine content they spread and comparing it with the effects of Russian trolls, whose messages primarily sought to increase discord online. Dyer [26] determined that after Russian trolls, bots were the most prolific vaccine-related tweeters. Huang and Carley [27] found that accounts linking to coronavirus information from less reliable sites were more likely to be bots. Ng and Carley [28] also found that bots change vaccine stance more easily than non-bots. Hence, understanding the actions of automated accounts is a crucial part of vaccine-related online influence campaigns.

#### The BEND Framework

A crucial component of social cybersecurity’s efforts to characterize online influence operations has been the struggle to establish the motives and tactics of those seeking to manipulate conversations in cyberspace. The BEND framework was developed to assist in the theoretical conceptualization of this problem by providing a taxonomy of 16 categories of maneuvers for conducting online influence [29]. These categories are divided into 2 types: narrative and network maneuvers. These types are further divided into positive and negative directions of influence. Narrative maneuvers focus on the information and content of messages. These maneuvers affect what is being discussed and how it is discussed. Network maneuvers focus on how the network and communities are shaped and the positions of key actors.

The BEND framework provides analysts and researchers with a way to conceptualize the tactics used in online information operations. Though this framework was discussed in reference to election manipulation [29], it has not yet been applied to vaccine-related influence campaigns.

## Methods

**Figure 1:**
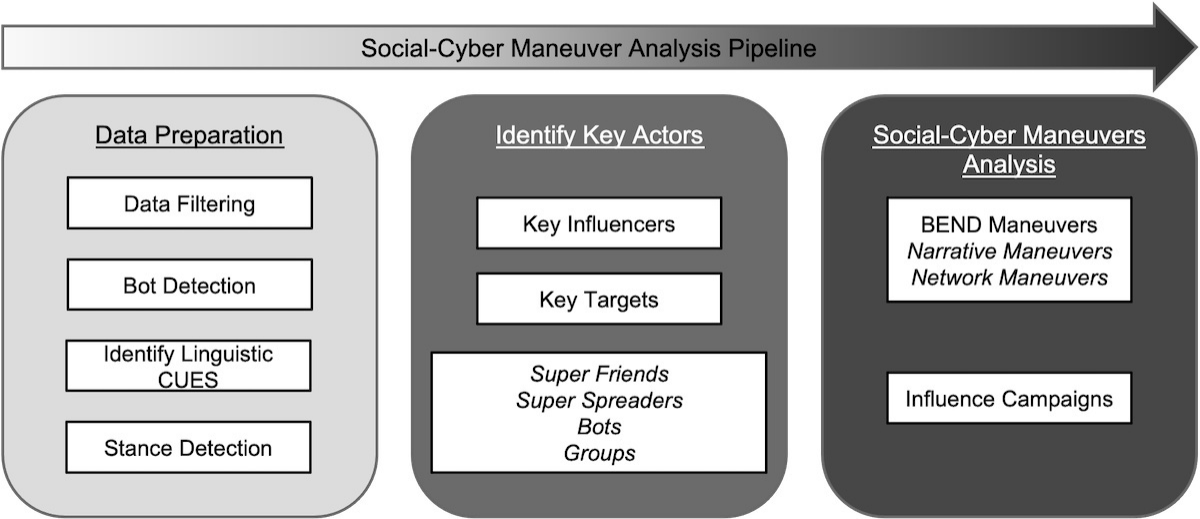
Social-cyber maneuver analysis pipeline.

In this work, we used a methodology similar to the pipelines in other social cyber-security studies [30]. For the social-cyber maneuver analysis, the end state is to gain a comprehensive understanding of the actors and their maneuvers used to manipulate others on social networks. The pipeline is broken into 3 parts: data preparation, key actor identification, and social-cyber maneuvers analysis (Figure 1).

### Data Preparation

#### Data Collection

The data used in this work are a subset of COVID-19 tweets collected from Twitter using the Twitter application programming interface (API) and keywords related to COVID-19. The data set was then further filtered using the vaccine-related terms shown in Table 1. Furthermore, we removed tweets from non-English speaking users from the data.

**Table 1:** Keywords used to collect COVID-19 vaccine-related tweets.

Filter	Keywords
Filter 1: COVID-19 tweets	coronaravirus, coronavirus, wuhan virus, wuhanvirus, 2019nCoV, NCoV, NCoV2019, covid-19, covid19, covid 19
Filter 2: vaccine tweets	vaccine, vax, mRNA, autoimmuneencephalitis, vaccination, getvaccinated, covidisjustacold, autism, covidshotcount, dose1, dose2, VAERS, GBS, believemothers, mybodymychoice, thisisourshot, killthevirus, proscience, immunization, gotmyshot, igottheshot, covidvaccinated, beatcovid19, moderna, astrazeneca, pfizer, johnson & johnson, j&j, johnson and johnson, jandj

We divided the data into 3 time periods surrounding the introduction of the Pfizer vaccine: December 1-7, 2020 (the week before the rollout), December 8-10, 2020 (during the week of the rollout in the United States and the United Kingdom), and January 25-31, 2021 (6 weeks after the rollout). The 3 periods consisted of 471,962, 694,200, and 662,776 users and 935,709, 1,511,344, and 1,368,035 tweets, respectively.

#### Identifying Bots

Using the Tier-1 BotHunter algorithm by Beskow and Carley [31,32], we determined the probability that each user within the data set was a bot. BotHunter is a random forest regression model trained on labeled Twitter data sets. It was developed from forensic analyses of events with extensively reported bot activity, such as the attack against the Atlantic Council Digital Forensic Research Lab in 2017. This machine learning model considers network-level features (such as the number of followers and friends), user-level attributes (including screen name length and account age), and tweet-level features (such as timing and content). For this work, any score of 75% or greater was labeled as a bot to reduce the chance of false positives and ensure that the accounts classified as bots were truly bots (at the expense of missing some bots) [33].

#### Linguistic Cues

The NetMapper software [34] was used to extract linguistic cues from the tweet text. These are metrics helpful in identifying a tweet's sentiment and author's emotional state [11]. Examples of these cues include the frequency of positive and negative terms, types of pronouns, emojis, and others. These tweet attributes are used to identify BEND maneuvers and actors participating in such maneuvers.

#### Organization Risk Analyzer - PRO Software

The Organization Risk Analyzer (ORA)-PRO software [34] is a dynamic meta-network analysis tool used extensively in this study to examine and characterize key actors, conversations, and the overall structure of the Twitter data. Key features used included a network data visualization tool, stance detection function, Twitter analysis report, and the BEND and Community Assessment report.

#### Stance Detection

We used the stance detector [35] built into ORA-PRO to divide the data set into the pro-vaccine and anti-vaccine communities. This stance detector starts with a set of hashtags that the user initially labels as pro- and anti- with respect to an issue. The stance detector uses these hashtags to label the stance of the Twitter accounts that used them. The algorithm then uses the concept of influence propagation to label the stance of users who did not use any of the pre-labeled hashtags. This propagation through the user communication network proceeds by repeating 2 steps. First, users with a known stance are used to determine the stances of some of the hashtags that have not yet been labeled. In this step, hashtags that are used overwhelmingly by users of one stance over the other are accordingly assigned that stance. In the second step, hashtags with a known stance are used to determine the stances of some of the unlabeled users. Users who have overwhelmingly used hashtags of one stance rather than the other are labeled with that stance. Additionally, both steps allow stance to spread directly from user to user. In both steps, unlabeled users who are predominantly connected to users of the same stance are assigned that stance.

The algorithm also provides a confidence level for each stance classification. After running the stance detector on our data, pro-vaccine users had a mean confidence level of approximately 99% to 100% for each time period. However, the anti-vaccine users for the before, during, and after rollout periods had mean confidence levels of 84%, 85%, and 67%, respectively. Table 2 shows the number of users classified by stance for each time period and the number of tweets by these communities. There were noticeably fewer anti-vaccine users and tweets than pro-vaccine users and tweets. Though the stance detector also identified neutral nodes, we excluded them from this study.

**Table 2:** The number of users labeled as pro-vaccine and anti-vaccine, along with the number of tweets by users of each stance after running the stance detector.

Time period	Users labeled by stance detection		Number of tweets by users of each stance	
	Pro-vaccine	Anti-vaccine	Pro-vaccine	Anti-vaccine
Before rollout	216,156	36,609	186,726	31,200
During rollout	195,334	47,566	292,607	55,406
After rollout	430,278	19,519	338,035	30,560

### Examining Key Actors and Social-Cyber Maneuvers

We used reports within ORA-PRO to gain insight on key actors, individual tweets, BEND maneuvers [36], and the entire network. We ran the reports and analyzed each of the 3 time periods on each of the subsequent stance communities. By examining an entire time period, we observed the interactions of the users between those of opposing or neutral stances. Then, by focusing on the individual communities by stance, we conducted a more fine-grained analysis.

ORA-PRO’s Twitter report allows us to identify and analyze key agents or actors, hashtags, tweets, and other Twitter attributes on Twitter data. Key actors are useful in understanding who are the most influential entities and what are the most influential conversations. The first type of key actors we observed was super friends, or users that exhibit frequent 2-way communication with others, such as reciprocal mentioning or retweeting. The second type that we examined was super spreaders. These users generate content that is shared often, facilitating the diffusion of information across the network. We then extracted the list of tweets and hashtags for each of these key actors for further inspection.

Additionally, the Twitter report identified valuable tweets. In this study, we specifically focused on the most propagated tweets within a data set. These are tweets that have the highest combined values for retweets, replies, and quotes. This information aided in understanding social-cyber maneuver narratives and actions.

The ORA-PRO software uses NetMapper's linguistic cues as input for detecting BEND maneuvers in tweets using the BEND and Community Assessment report. Of the most propagated tweets, we used this report in conjunction with manual labeling to gain insight into the social-cyber maneuvers used within our data sets.

In our analysis, we organized the BEND maneuvers into 1 of 6 application categories based on the similarity of the maneuvers: developing the narrative, emotional influence, countering the narrative, affecting leaders, making or growing groups, or dissolving or reducing groups (Table 3). These represent macro-level actions occurring as a result of multiple BEND maneuvers. We observed these combinations over time to identify a concerted effort to influence target audiences of their stances. We labeled the narratives and actions for the 100 most propagated tweets for each stance community within each time period and grouped them into these application categories.

**Table 3:** BEND maneuvers organized into application categories.

BEND maneuver and application categories			Maneuvers
**Narrative maneuvers**			
	Developing narrative	Engage, explain, enhance	
	Emotional influence	Excite, dismay	
	Countering narrative	Distract, dismiss, distort	
**Network maneuvers**			
	Affecting leaders	Back, neutralize	
	Making groups	Build, boost, bridge	
	Reducing groups	Neglect, narrow, nuke	

## Results

### Key Actor Overview Overtime

#### Key Influencers: Super Friends and Super Spreaders

We used ORA-PRO to calculate the super friends and super spreaders for the 3 data sets for each time period and identified the types of entities that fell into each of these categories.

The top 10 super friends throughout the 3 data sets were predominately pro-vaccine, though varied in the types of actors. All of the top 10 super friends identified before the rollout were unverified Twitter accounts and relatively low-profile users. ORA classified all of the tweets as pro-vaccine, and 3 of them, we identified as amplifier bots [37]. During the rollout, the top 10 included several anti-vaccine users and a single neutral stance user. Of the 3 bots on the list during this period, 2 news bots emerged alongside one of the pro-vaccine bots from the before period. At 6 weeks later, several higher-profile users from health and government organizations appeared as super friends. These included the World Health Organization, the India Ministry of Health, and the India Official COVID Response account.

Except for one instance, the top 10 super spreaders were either classified as pro-vaccine or neutral within the 3 data sets. All of the users before and during the rollout were high-profile verified Twitter accounts. During these 2 periods, the super spreaders were primarily health organizations, vaccine manufacturers, news organizations, and senior government leaders. After the rollout, the types of accounts identified as super spreaders changed. Though a couple of news organizations and health-related accounts remained on the list, the users were more community leaders or professionals with a substantial reach, such as actors or journalists.

#### Bot Influencers

**Figure 2:**
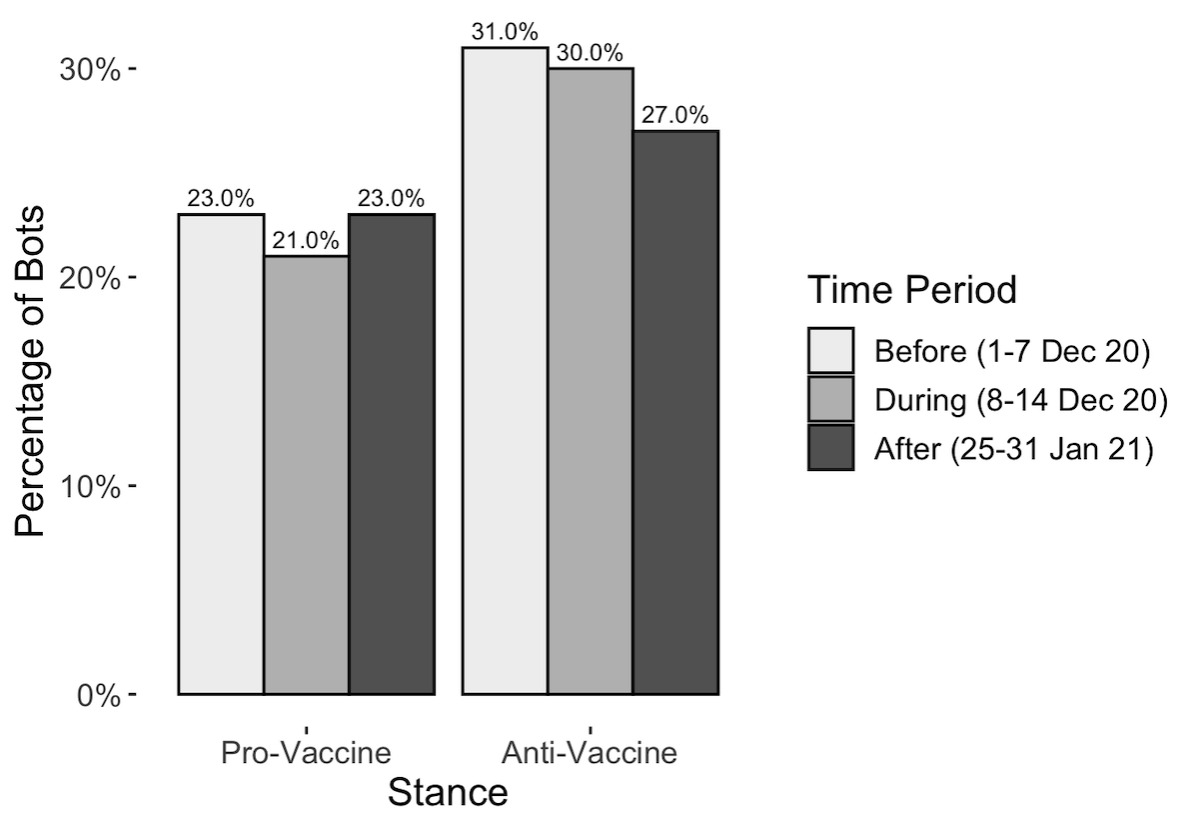
Percentage of bots by stance by time period.

The BotHunter results revealed that anti-vaccine agents consisted of a higher percentage of bots than the pro-vaccine agents (Figure 2). Though anti-vaccine bots decreased over time, the number of bots remained relatively higher than the total percentage of pro-vaccine bots of the same time periods.

**Figure 3:**
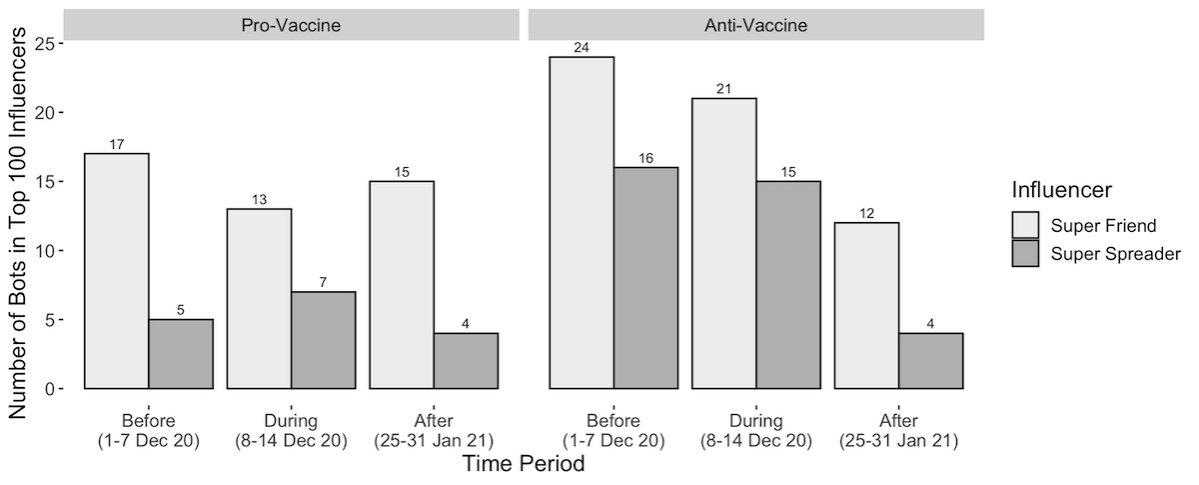
Number of bots in top 100 influencers.

We calculated the number of bots within the top 100 super spreaders and super friends (Figure 3). The high percentage of super friends shows that users are interacting with the bots and engaging in 2-way communications. The super spreaders show that bots are effectively diffusing tweets through the network. These bots have managed to connect with users on Twitter, which makes them susceptible to the information or disinformation that these bots can be posting. Furthermore, the data show a noticeable decline in the number of anti-vaccine influencers after the rollout. Super spreaders, for example, reduced from 15 and 16 bots during the first 2 periods to only 4 after the rollout. This difference is likely a result of the Twitter policy against anti-vaccine disinformation enacted mid-December 2020 [9].

### BEND—Narrative Maneuvers

Using ORA-PRO, we identified the most propagated messages for each of the stances and periods. We then identified and evaluated how narratives manifested themselves as maneuvers to persuade others. This section provides a cumulation of narrative BEND maneuvers observed within each of the communities.

#### Pro-Vaccine Communities—Narrative Maneuvers

Pro-vaccine communities had varying messages before the vaccine rollout. Many of them were *excite* messages, defined as messages that elicit a positive emotion such as joy or excitement. Users posted positive tweets about the vaccine’s approval and then encouraged others to get their vaccine when it became available. At the same time, many users also attempted to compel others to curb the growing number of COVID-19–related illnesses and deaths using the *dismay* maneuver. This is messaging to elicit negative emotion such as sadness or anger, to warn users of the consequences of not getting the vaccine. Health officials and organizations used the maneuver, *explain*, which is to educate on a topic using details and relevant facts, to inform the science behind the vaccine and build confidence in its use. To counter vaccine myths, users used the *dismiss* maneuver, the maneuver used to downplay anti-vaccine information as either irrelevant, inconsequential, or foolish. Many users also *dismissed* many of the fears that stemmed from the vaccine’s rapid development. These maneuvers were typically followed up with *explain* maneuvers that attempted to debunk these myths using scientific evidence or detailed forms of justification. Finally, users would *enhance* their pro-vaccine ideas or encourage their views with the support of prominent actors or interesting content. Many, for example, tweeted and quoted articles about 3 former US Presidents volunteering to get the vaccine to promote trust in the vaccine.

During the rollout, pro-vaccine communities continued to post similar types of messages as in the week prior. Many users expressed *excitement* about the first person to receive the Pfizer vaccine, a 90-year-old woman from the United Kingdom leading the vaccine rollout. Other types of optimistic *excite* messages included those from users of various countries approving and purchasing vaccines as well as many *excited* at the sight of the logistics vehicles containing the vaccines within the distribution process. Additionally, pro-vaccine proponents also added to their *explain* messages about how the vaccines work by emphasizing the vaccine’s effectiveness after the first dose and supporting the overall narrative with charts and results from the vaccine trials during the development process. Furthermore, medical professionals made efforts to *engage* with their more hesitant audiences to instill confidence in the vaccine and encourage them to get vaccinated.

After the rollout, messages continued to *explain* science-backed research for the development, safety, and efficacy of the vaccine to build trust in its use while countering anti-vaccine myths and narratives. Pro-vaccine users during this period showed general *excitement* and optimism about how the vaccine will benefit themselves, their families, and their communities. There were general *excite* tweets about the authorizations and distributions of the vaccine worldwide. Individuals also spread *excite* messages about finally getting the vaccine, getting an appointment for the vaccine, or just desiring to get the vaccine. Many users combined these messages with the *engage* maneuver by taking ownership of the vaccination process by setting the example as a vaccinated individual and encouraging others to also get vaccinated.

Throughout these periods, pro-vaccine communities engaged in hashtag hijacking by tying pro-vaccine narratives to hashtags intuitively associated with anti-vaccine messages. By adding #antivax, #antivaxxer, and other similar anti-vaccine-related hashtags to their tweets, pro-vaccine communities used these hashtags in large numbers to draw attention to pro-vaccine messages with anti-vaccine keywords (Table 4). In one case, they used it to *enhance* the pro-vaccine messages, typically by attaching this hashtag to pro-vaccine *explain* messages intended for vaccine hesitant users. In another case, hashtag hijacking tied the hashtag to satirical messages related to anti-vaccine individuals’ actions. The pro-vaccine message *distorts* the anti-vaccine message with a quote or a reply or somehow ties their narrative to a specific anti-vaccine incident. Furthermore, in some uses of the hashtags, pro-vaccine users *engaged* anti-vaccine users to condemn or insult them for either spreading disinformation or other anti-vaccine behavior.

**Table 4:** Hashtag hijacking: usage count of anti-vaccine–related hashtags by pro-vaccine users.

Hashtag	Before rollout (n=2218), n	During rollout (n=1221), n	After rollout (n=768), n
antivaccination	0	26	5
antivaccine	55	47	68
antivax	457	281	247
antivaxer	5	3	0
antivaxers	26	11	13
antivaxx	83	54	63
antivaxxer	133	39	62
antivaxxers	1459	760	310

#### Anti-Vaccine Communities—Narrative Maneuvers

In the week leading up to the vaccine approval and distribution, users were already expressing their COVID-19 anti-vaccine views on social media. The most popular types of messages were the emotionally appealing *dismay* messages about side effects from the vaccine. Anti-vaccine users shared messages about how the vaccine causes female infertility, destroys the immune system, or leads to death. These messages were further *enhanced* with references to scientists, doctors, former Pfizer representatives, and politicians. In many of these messages, the side effects were *explained* using plausible arguments and pseudoscientific methods and information. To counter pro-vaccine messages, anti-vaccine proponents attempted tactics such as *dismissing* the vaccine effectiveness, suggesting that a person’s immune system is more than sufficient against the virus. They also countered with the *distract* maneuver, which uses misdirection by making other topics seem more important. In one example, the 3 US Presidents volunteering for the vaccine mentioned earlier sought to build confidence. However, opponents of the vaccine focused on *distracting* their audiences with negative political news from the Presidents’ pasts relations with China. These insinuated negative links between the Presidents and the country where the virus first began to spread. In another narrative, messages specifically targeting pro-life supporters described the use of fetal cells derived from an abortion during the vaccine development process. These began as *dismay* messages to anger pro-life supporters about its use and then was supported with *explanations* on how the different vaccines used the fetal cells in different phases of the process, making some vaccines more ethical than the others. Proponents then *enhanced* these messages by attaching supportive messages from major religious organizations.

During the rollout, anti-vaccine narratives continued to emphasize many of the negative aspects of the vaccine. Still, many *dismaying* messages about the vaccine side effects dominated anti-vaccine conversations. New *explaining* messages to support these *dismaying* claims included citing the vaccines’ published lists of adverse effects and a cost-benefit analysis on the benefits versus the severe reactions resulting from getting the vaccine. Again, these were *enhanced* and validated with statements from medical professionals and scientists. Additional *dismaying* messages emerged as popular during this early period. Topics included news reports for the vaccine causing false positives for HIV, government cautions for allergic reactions to the vaccines, claims that the vaccine is not Halal certified under Islamic dietary laws, and negative experiences from those who participated in the vaccine trials. *Distort* messages, or discussion that alters the main message, helped anti-vaccine messages counter many positive pro-vaccine narratives and propagate anti-vaccine conspiracy theories. They countered the scientific facts about the construct of the vaccine with the lack of peer-reviewed literature to support it, spread manipulated images of Dolly Parton purporting that the vaccine caused her to have Bell’s palsy, and suggested that the mRNA vaccines contain nanobots and can change a person’s DNA. Furthermore, general anti-vaccine messages from both medical and nonmedical users within this community *engaged* online to express their distrust with the vaccine and recommend not getting the shot without knowing the long-term safety data.

After the rollout, the decrease in anti-vaccine users resulted in spreading fewer anti-vaccine messages. By this time, Twitter removed many anti-vaccine users and their messages for violating their policy on spreading false or misleading COVID-19 vaccine information. The messages that remained, however, were still primarily *dismaying* and *distorting* messages about the adverse side effects and deaths resulting from vaccinations. Despite the Twitter policy, several *distorting* conspiracy theories such as vaccines connecting one’s body to cryptocurrency and altering DNA still appeared in the data set. Furthermore, users continued to *engage* their audiences more practically by expressing hesitancy for a quickly developed vaccine without data on its long-term effects.

### BEND—Network Maneuvers

Using the messages, we identified instances of the communities engaging in network maneuvers. Network maneuvers alter the structure of the network by encouraging connections or disconnections between users. In Twitter, one effective tool and indication of a network maneuver is the use of mentions. These types of maneuvers, however, can exist without them.

There were several ways that pro-vaccine communities engaged in network maneuvers. The most common maneuvers were *building* and *boosting*, used for creating a group or to grow the size of a group, respectively. The primary goal for pro-vaccine communities was to urge others to get the vaccine under the premise that the more people who supported and received the vaccine, the sooner the pandemic would end. Simultaneously, these groups engaged in the counter maneuver of *narrowing* and *neglecting* to reduce the size of or marginalize the opposing anti-vaccine community. One of the most effective actions for group reduction was using the *nuke* maneuver to dismantle or show the appearance of a dismantled anti-vaccine community. Twitter attempted this maneuver when it created its policy against COVID-19 vaccine disinformation, affecting the entire after-rollout period data set. Another common network maneuver was *backing*, which is an action that increases the importance of leaders or creates new leaders. Pro-vaccine users showed support for government officials, leaders in the medical field, health organizations, and vaccine manufacturers with positive messages and references to these leaders or organizations.

The anti-vaccine community conducted similar network maneuvers to the pro-vaccine community. They aimed to *build* and *boost* their group and reduce the pro-vaccine community using *narrowing* and *neglecting*. This community, however, did not have as many leaders as its opponents. The few that they *backed* included critics of pro-vaccine policies such as an ex-Pfizer vice president, politicians, and scientists who petitioned against the vaccine for safety concerns. Anti-vaccine users, however, had a large selection of leaders that they attempted to *neutralize* or decrease in importance. These opposing leaders were largely the same people and organizations the pro-vaccine community *backed*.

### Social-Cyber Maneuvers Applications Over Time

The different narratives and BEND maneuvers from each stance community were associated with one or more application categories. Though each community used different content for their messaging, they used roughly the same techniques. Many of these techniques are used in combination over time to develop more impactful influence campaigns.

**Figure 4:**
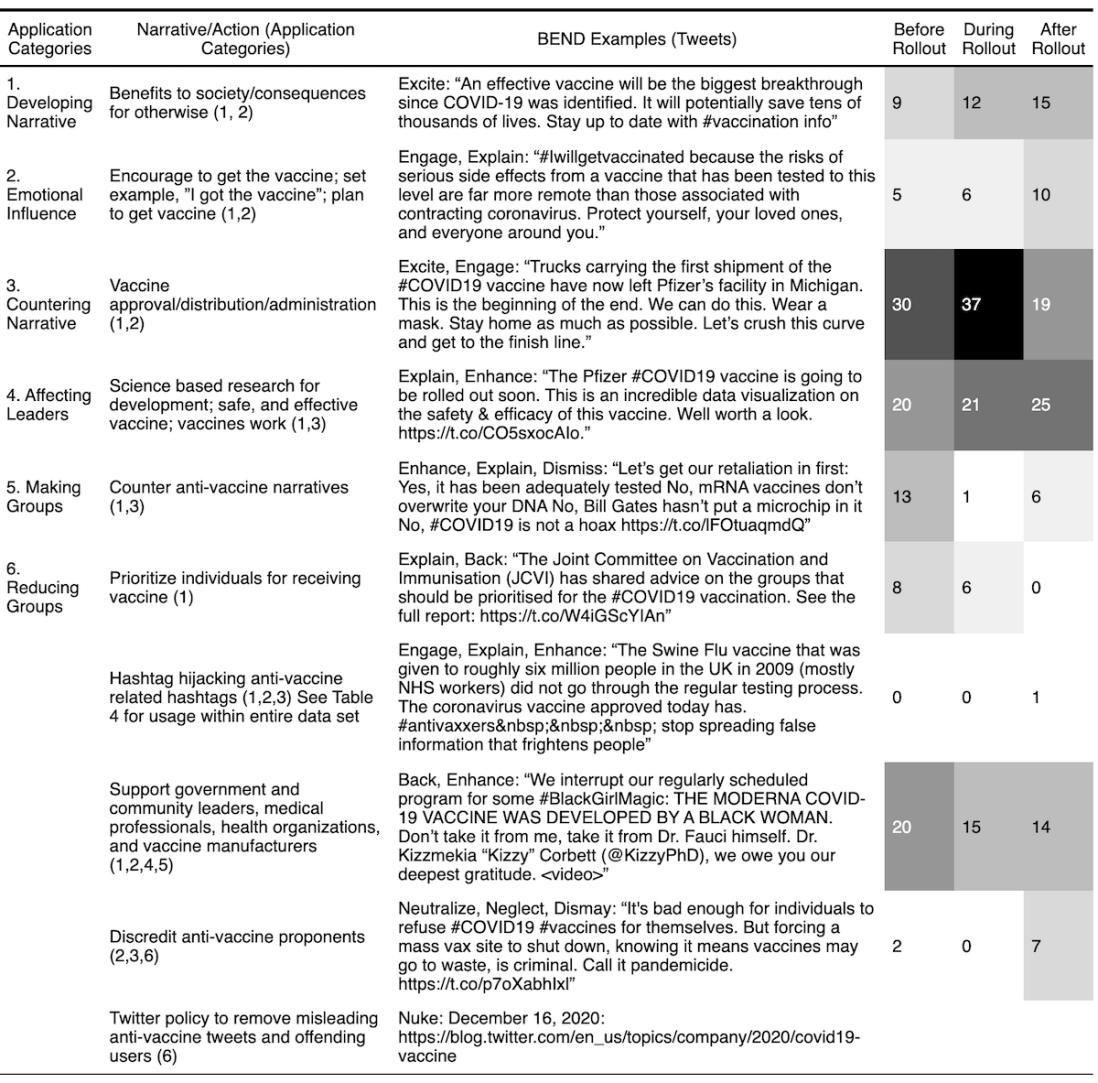
Social-cyber maneuvers and narratives for top 100 most propagated pro-vaccine tweets.

Over time, pro-vaccine communities consistently used mostly positive narrative content in their messaging while applying a pattern of developing their narrative, using emotional influence, and countering the opposing community’s narrative (Figure 4). *Excite* messages combined with the narratives about the approval, distribution, and administration of the vaccine were among the highest types of propagated tweets among the 3 periods. The second highest categorization of tweets developed the narrative of creating the vaccine using science-based research to build confidence in its safety and effectiveness. This narrative was also used as content to counter anti-vaccine narratives that were often fake or pseudoscience. Furthermore, we found that directly countering anti-vaccine narratives became less common over time as the number of anti-vaccine messages decreased and the primary pro-vaccine narratives became prevalent.

Because the anti-vaccine community did not have prominent leaders to *neutralize*, the pro-vaccine community regularly focused on *backing* the leaders within their own community, such as government officials, health organizations, and vaccine manufacturers. The pro-vaccine community also attempted to expand their group by using hashtag hijacking. Though few instances occurred within the top 100 most propagated tweets, hashtag hijacking still occurs throughout the data set, as shown in Table 4. Finally, although there were many attempts to reduce the anti-vaccine community through varying narratives, Twitter’s policy to counter anti-vaccine disinformation before the last time period created the most apparent change in network structure. The result was the decrease in accounts that typically propagated offensive disinformation and subsequently large amounts of anti-vaccine tweets.

**Figure 5:**
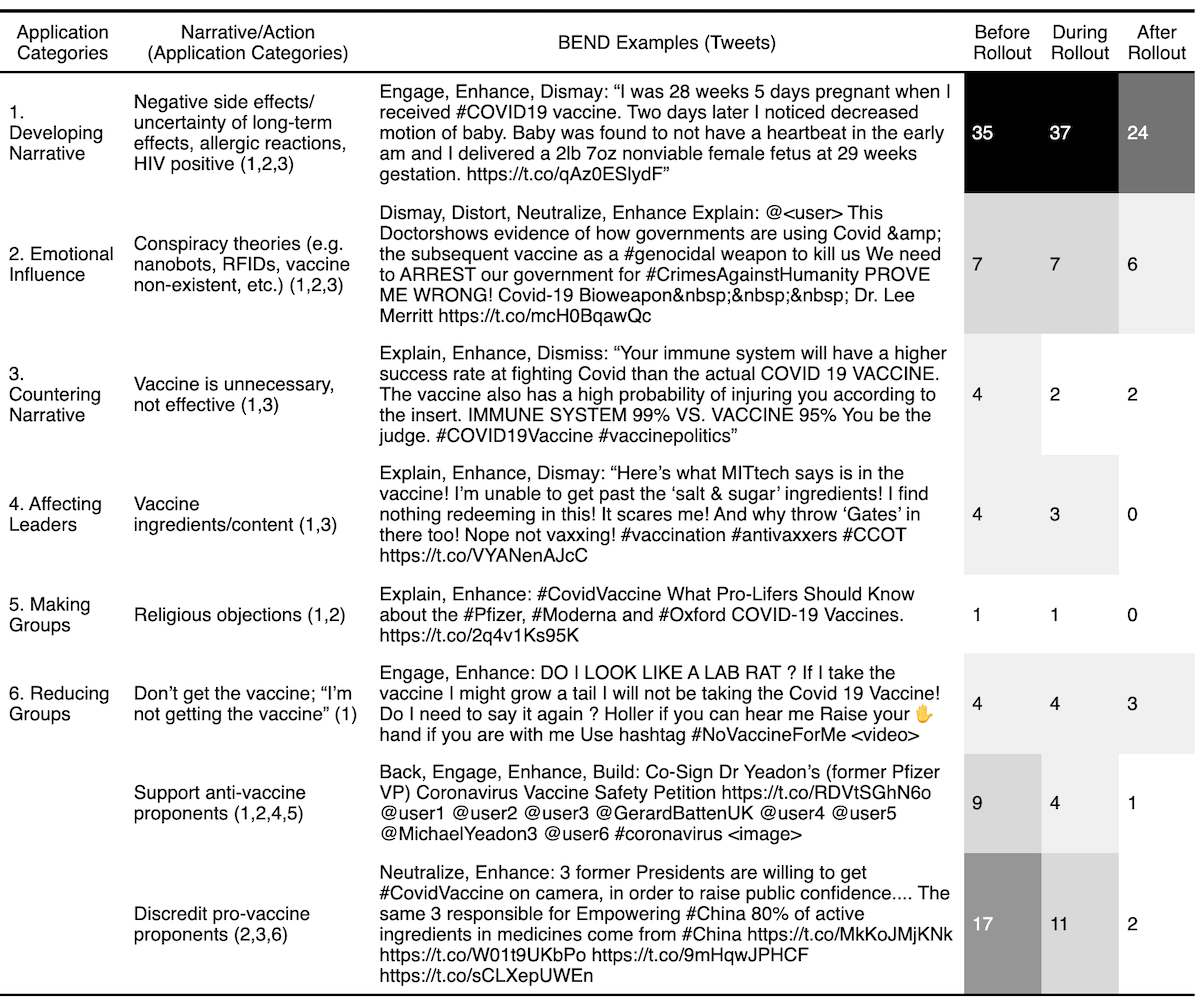
Social-cyber maneuvers and narratives for top 100 most propagated anti-vaccine tweets.

Across the 3 time periods, the anti-vaccine community also used different combinations of the maneuvers and applications to sway their audience (Figure 5). They primarily developed their narrative and countered pro-vaccine messages using multiple maneuvers, heavily relying on the negative emotional influence using *dismaying* messages to highlight the side effects, long-term effects, and conspiracy theories. Anti-vaccine users also aimed to affect the relationships of leaders of both communities. They aimed to discredit the leaders of the pro-vaccine community while highlighting the negative messaging of medical professionals and scientists within their own community. During the first 2 periods, these themes appeared consistently. However, after the rollout following the removal of anti-vaccine accounts and messages, negative side effect type of messages emerged as the dominant narrative. Because of the decrease in messages, it would be difficult to speculate about the types of anti-vaccine maneuvers that may have otherwise prevailed during the later period.

## Discussion

### Principal Findings

The results in this study showed the differing characteristics of pro-vaccine and anti-vaccine communities on Twitter and how they manipulated narratives and the online network structure to convince users whether to vaccinate against COVID-19. Both groups of users used many of the same approaches but varied in the extent to which they applied each maneuver. They sought to *build* their communities while, at the same time, attempting to reduce the size of the opposing community by maneuvering as appropriate to fit their narratives. Pro-vaccine supporters tweeted *excite* and *explain* messages to encourage vaccination, whereas anti-vaccine users relied on the negative *dismay* and *distort* messages with narratives related to side effects and death. Pro-vaccine users also *backed* the prevalent leaders within their group, of whom anti-vaccine users targeted and attempted to *neutralize*. Furthermore, platform policies showed their ability to effectively *nuke* the anti-vaccine community by reducing the size of the online community and the quantity of anti-vaccine messages.

The majority of top super spreaders and super friends for each of the analyzed time periods were pro-vaccine users. Government leaders, medical organizations and professionals, vaccine manufacturers, and, in the later period, less-mainstream community leaders emerged as pro-vaccine leaders, effectively reaching a higher number of users and engaging in more 2-way conversations. Additionally, bots had a sizeable presence within each community. We observed a larger percentage of bots within the anti-vaccine community than the pro-vaccine community, and among the top 100 key influencers for each community over time, the anti-vaccine community had more bots as super spreaders and super friends. Before Twitter’s vaccine disinformation policy, they reached as high as 24% and 16% of the top 100 anti-vaccine super friends and super spreaders, respectively, before the rollout. The anti-vaccine community utilized bots to a greater extent to *build* their communities and spread their narratives than did the pro-vaccine community, effectively positioning them as key influencers among anti-vaccine users.

Pro-vaccine users repeated many of the same maneuvers throughout each time period, varying in different narratives that emerged as the rollout occurred. Many of the maneuvers were positive or growth-type maneuvers. They developed narratives around science-based facts *explaining* the safety and effectiveness of the vaccine and emotionally influencing narratives that *excited* their audiences about the health and societal benefits of everyone receiving the vaccine and, to a lesser extent, *dismayed* them with the fatal consequences of not vaccinating. Many of the topics used to develop these narratives were also used to counter anti-vaccine messages. As many anti-vaccine conversations revolved around side effects and conspiracy theories, pro-vaccine proponents rebutted with facts to *explain* the errors in these messages. These narratives were used consistently by highly connected leaders within the community and actors that maintained a high profile apart from Twitter, such as government leaders, news organizations, and medical professionals. Pro-vaccine users tended to *back* government officials and health organizations with positive messaging to build confidence in the proponents of the vaccine as well as the vaccine itself.

Throughout each of the time periods, the anti-vaccine community used similar maneuvers to those the pro-vaccine community used but with a greater frequency of the negative or reducing-type maneuvers. They developed focused narratives and countered pro-vaccine messaging to increase hesitancy and doubt. Their most consistent technique was using *dismaying* messages of the adverse side effects, the uncertainty of the long-term effects, and vaccine deaths. Conspiracy theories about the vaccine also added to the anti-vaccine narrative. Users attempted to *neutralize* pro-vaccine leaders by discrediting them and their associated messaging, and they *backed* leaders who criticized the vaccine and encouraged others not to get the vaccine.

Finally, as the host for the pro-vaccine and anti-vaccine engagements, Twitter is in a unique position with the ability to filter the discussion on their platform. Their policy to remove misleading and false anti-vaccine *nuked* the anti-vaccine community by significantly reducing the number of anti-vaccine users and tweets that had grown at the time of the rollout. The social media site made a policy to fight disinformation, which resulted in supporting the pro-vaccine effort to reduce the size of the anti-vaccine community and messaging.

### Limitations

One major limitation was the ability for the stance detection to separate the nodes into pro-vaccine and anti-vaccine communities. First, many pro-vaccine maneuvers use neutral hashtags. Second, anti-vaccine mean confidence levels for each time period were lower than those for pro-vaccine, with the after-rollout data only reaching as high as 67%, even after multiple iterations of hashtag labeling. Third, hashtag latching made stance selection difficult as some hashtags commonly used for pro- or anti-vaccine messages were used to gain the attention of members of the other community. Therefore, further study is required for improving the separation between pro- and anti-vaccine agents and tweets.

A second limitation is the ability of ORA-PRO to detect BEND maneuvers. This required manual verification of select entities within the data set. Newer versions of ORA-PRO continue to refine the metrics to better identify some of the maneuvers, especially network maneuvers, that occur over time. The results of this study inform the specifications and thresholds for improving the software.

Finally, many tweets and users that existed during the initial data collection were either deleted or suspended due to Twitter Rules violations. This made it difficult for observing historical tweets with their associated images and videos as well as visualizing tweets with replies and mentions within the Twitter environment.

### Conclusions

In this paper, we analyzed how pro-vaccine and anti-vaccine communities around the initial COVID-19 vaccine administration attempted to persuade others of their stance under the BEND maneuvers framework. The BEND maneuvers allowed us to examine the different techniques used by each community. We observed the actions of different types of key actors within the different groups and analyzed their varying techniques. Additionally, we examined the main concepts and messages of tweets tweeted within each community and the extent they acted as each of these social-cyber maneuvers. These maneuvers were combined into application categories to gain a macro-level understanding of how the maneuvers were used in combination as an overarching influence campaign over time.

Real world events influence online discussions, and over time, the changes in these conversations reflect changes in beliefs. In this case, the efforts of these 2 communities can lead users to either vaccinate themselves against COVID-19 or not, possibly changing the direction of the pandemic. Future work should look at how these changes in beliefs mobilize into changes in behavior. Furthermore, though many influencing actions result from users interacting with other users, the policies that govern the use of social media can impact the size of a community and their ability to spread their narrative throughout the network. Therefore, research to regularly detect and evaluate the effectiveness of social-cyber maneuvers and make pointed network structure alterations based on specific narratives is needed to understand the consequences of different interventions and implement better policies to impact influence campaigns on social media.

## Abbreviations

API: application programming interface
BERT: Bidirectional Encoder Representations from Transformers
E/I Index: external/internal index
EMA: European Medicines Agency
FDA: Food and Drug Administration
ORA-PRO: Organization Risk Analyzer - PRO
